# Potassium-rich brine deposit in Lop Nor basin, Xinjiang, China

**DOI:** 10.1038/s41598-018-25993-6

**Published:** 2018-05-16

**Authors:** Ming-guang Sun, Li-chun Ma

**Affiliations:** MLR Key Laboratory of Metallogeny and Mineral Assessment, Institute of Mineral Resources, CAGS, Beijing, 100037 China

## Abstract

Lop Nor potash deposit is the largest sulfate-type liquid potassium salt deposit in China, consisting of three areas: Xinqing platform, Luobei depression, and Tenglong platform. In this study, the geological background, basin structure, and fracture system of the deposit, along with the brine storage size of the three ore bodies are introduced in detail, using underground brine elevation models of the mining areas. We collected 91 samples of brine from long-term observation boreholes in the Lop Nor ore district, and analyzed their ion contents. The KCl content of the brine varies from 1% to 1.45% and that of B_2_O_3_ varies from 1900 to 4500 mg/L, which are higher than the cut-off for Chinese industrial mining grades. The spatial distributions of KCl and B_2_O_3_ contents in different ore beds were plotted, and the distributions of different grades of K^+^ and B^3+^ were revealed.

## Introduction

Global potash resource reserves are rich, estimated at 250 billion tons (K_2_O)^1^, but most are subsurface solid minerals of marine origin. Although potassium resources are abundant, their distribution is uneven. Potash deposits are mainly found in Europe, North America, Central Asia, and Southeast Asia. China’s potash resources account for only 1.8% of the world’s potassium reserves^2^. China is also a large agricultural country, where fertilizer application has been ranked first in the world. Potash fertilizer consumption in China has reached 10 million tonnes per year. In 2014, China’s potash production was 4,300,000 t^3^, leaving a large gap between output and consumption, which although increases the value of domestic potash resources also resulted in the importation of large quantities of potassium resources.

**Figure 1:**
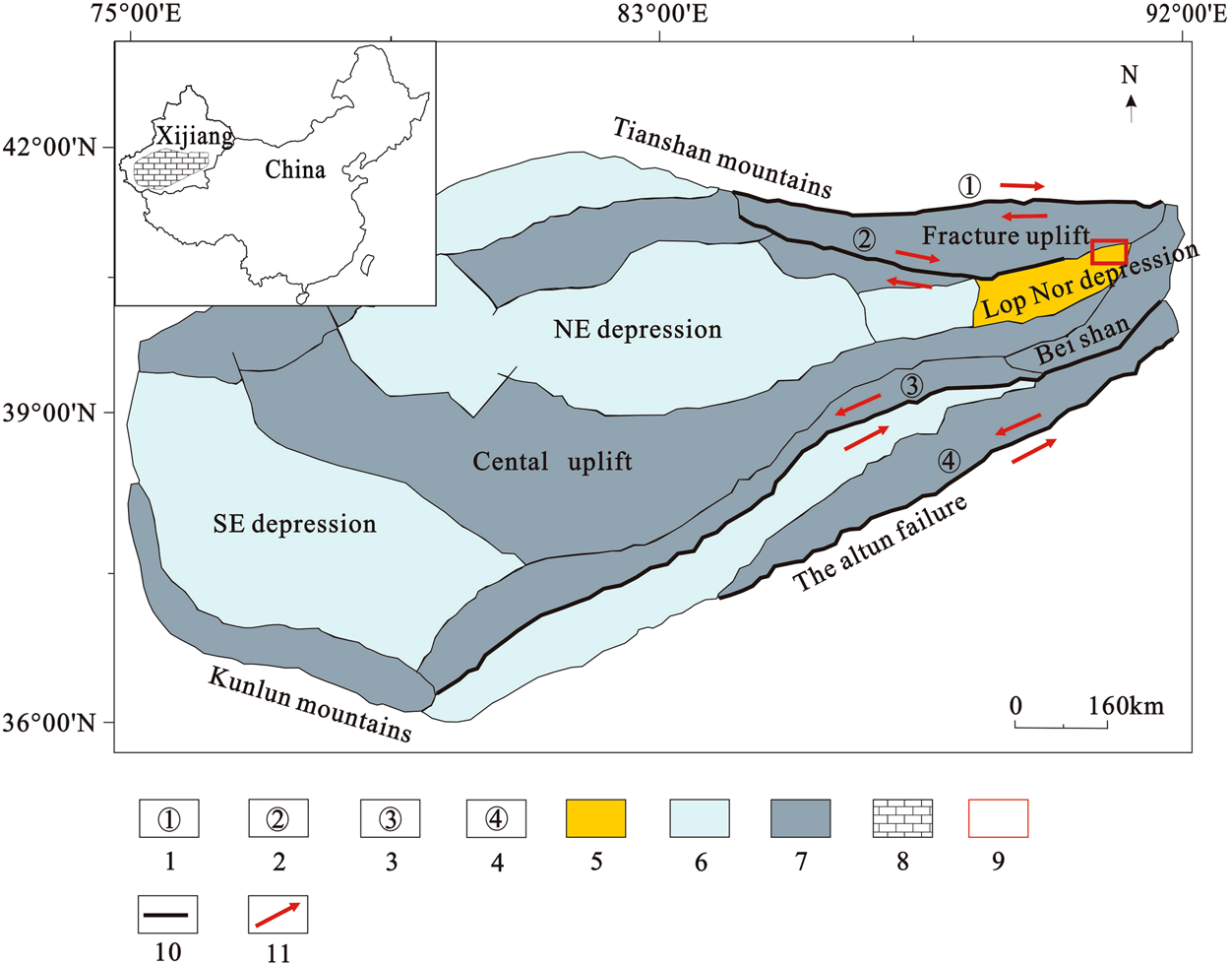
Structural map of the Tarim Basin in Xinjiang, China^21^. 1. Xing Di Dextral fracture, 2. Konqi River Dextral fracture, 3. Cheerchen Sinistral fracture, 4. Altun Sinistral fracture, 5. Lop Nor sub-basin, 6. Depression, 7. Uplift, 8. Tarim Basin, 9. Research area, 10. Fracture, 11. Strike-slip directions.

Solid potassium deposits are only found in the Mengyejing potassium deposit in Yunnan, China, with proven KCl reserves of 16.76 Mt^4^. Total proven reserves of potash in China are 0.99 Gt (KCl)^5^, of which brine type potash resources account for more than 98% of the total. Brine deposits are mainly distributed in the Qaidam Basin of Qinghai and the Lop Nor Basin in Xinjiang. The Lop Nor Basin is a Quaternary dry saline lake, located in a low-lying area at the eastern edge of the Tarim Basin in Xinjiang, with coordinate ranges of 39° to 41°N and 88° to 92°E (Fig. 1A). Since the 1995 discovery of the Lop Nor potassium-rich brine deposit, the amount of potassium chloride resources has been proven up to 145 Mt^6^. The Lop Nor deposit is the second largest brine potash deposit after that of the Qaidam Basin in Qinghai, China. Previous studies have been done on glauberite (CaSO_4_·Na_2_SO_4_) reservoir characteristics of brine potash deposits at Lop Nor^7^, chemistry of the potassium-rich brine^8–10^, and brine mining technology^11–15^, but studies of spatial distribution and geochemical characteristics of the Lop Nor deposit are less common. This paper introduces metallogenic geological background and distribution characteristics of ore bodies in the Lop Nor potassium-rich deposit. The spatial distribution of ore bed geochemistry and spatial enrichment trends of K and B are introduced through brine data from 91 drill holes, providing a basis for development and use of potash deposits in the Lop Nor basin.

## Research Area Overview

The Lop Nor basin is a secondary fault depression tectonic basin in the eastern Tarim Basin^16^, Central Asia’s largest inland basin. Its development was controlled by different periods of tectonism. At the end of the Neogene, Himalayan movement caused the western Tarim Basin to uplift and the eastern part to sink, tilting the terrain from southwest to northeast as the elevation decreased from 1400 m to 800 m. Lop Nor is located at the lowest elevation in the basin, also known as the Lop Nor depression. This is why Lop Nor has become a catchment area, providing abundant source material for brine deposit formation. The Lop Nor area experienced complex tectonic movements from the Cretaceous to the Tertiary^17^, and is now surrounded by mountains on three sides (Fig. 1), which create a closed hydrological environment for brine deposit formation. The region has an arid continental climate and is one of the driest regions in the world, with annual rainfall of less than 20 mm and annual evaporation of approximately 3500 mm^10^. The annual average daily air temperature is 11.6 °C, with the highest temperatures reaching 50 °C and daily temperature differences of up to 25 °C. These high temperatures and long periods of daylight are beneficial to the formation of evaporite minerals^18^ and are also favorable conditions for Lop Nor to become a salt deposition center. In this area, the prevailing winds are mainly from the east and northeast^19^. Average annual wind speed is 5.5 m/s, with maximum wind speed of up to 45 m/s, so wind erosion is strong and evidence can be seen everywhere of Quaternary erosional landforms called Yadan. The combination of structure, climate, and source supply in the Lop Nor area make the basin the largest production area for brine-type potash in China.

## Research Techniques

### Sampling method

**Figure 2:**
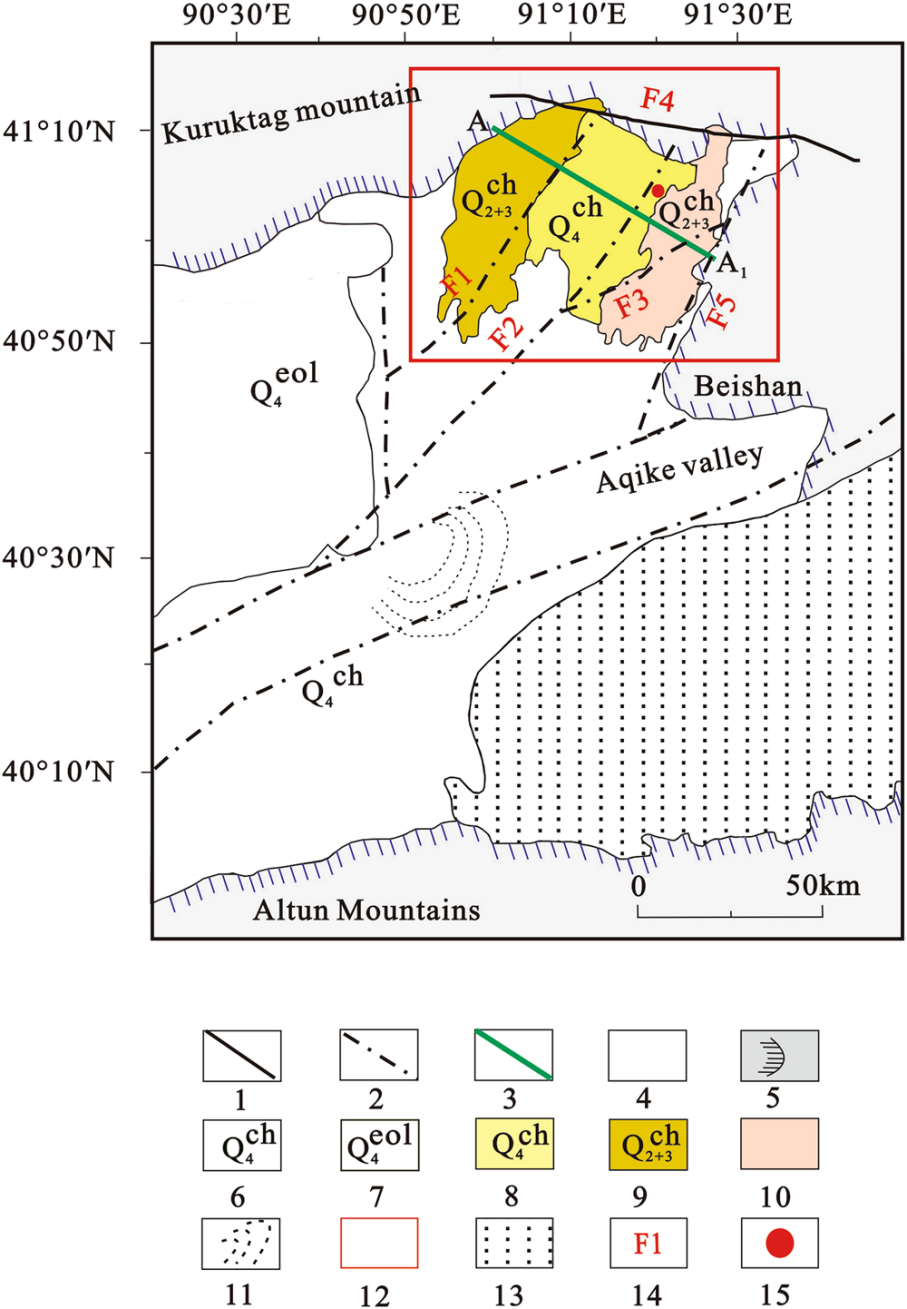
Distribution map of mining areas^23^. 1. Fracture; 2. buried fault; 3. section line; 4. Quaternary period; 5. pre-Quaternary; 6. Quaternary chemical deposition; 7. Quaternary aeolian deposits; 8. Luobei depression; 9. Xinqing platform; 10. Tenglong platform; 11. “Great ear” area; 12. research area; 13. desert; 14. main fractures; 15. ZK1200 drill hole.

**Figure 3:**
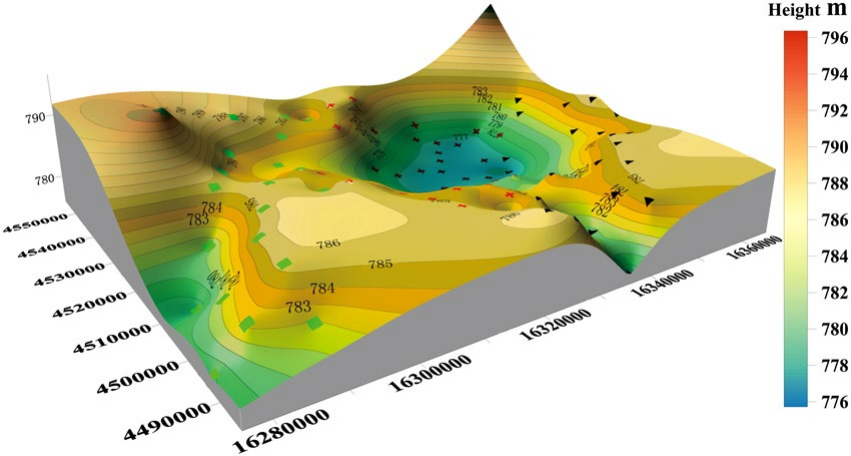
Ore district brine elevation and sampling point distribution map. In Fig. 3, the red crosses represent sampling points in the Luobei depression; the green squares represent sampling points in the Xinqing platform; and the black triangles represent sampling points in the Tenglong platform.

The Lop Nor potassium-rich brine deposit consists of three mining areas—the Tenglong platform, Luobei depression, and Xinqing platform. In July and August, 2016, we collected brine samples from 22 drill holes in Tenglong platform, 43 in the Luobei depression, and 26 in the Xinqing platform. *In situ* measurements of brine burial depth, density and pH were made; GPS was used for geolocation and elevation measurements. Two sample bottles (white plastic) were used to collect 500 ml of brine per hole. To prevent evaporation of the brine in the extreme arid climate or leaks during transportation, bottles were sealed quickly with tape after sampling. Locations of the three mining areas are shown in Fig. 2, and the sampling points are shown in Fig. 3.

### Analytical and mapping methods

Main elements Na^+^, K^+^, Mg^2+^, Ca^2+^, Cl^−^, SO_4_^2−^, CO_3_^2−^, and HCO_3_^−^ and trace elements Li^+^, B^3+^, Br^−^, I^−^, Rb^+^, Cs^+^, and Sr^2+^ were tested and analyzed in the brine samples. Na^+^, K^+^, Mg^2+^, Ca^2+^, Sr^2+^, Rb^+^, Li^+^, Cs^+^ were determined by atomic absorption spectrophotometry (error less than 2%). Br^−^, I^−^, CO_3_^2−^, HCO_3_^−^, and Cl^−^ were tested by conventional titration (error less than 5%). SO_4_^2−^ and B^3+^ were tested by visible light spectrophotometer (error less than 1%).

Sampling point location maps, elevation contour maps of the mining areas, and geochemical spatial distribution maps of brine in different ore beds were undertaken with Surfer 11 software^20^.

## Results and Discussion

### Mining area structures

The Lop Nor basin is situated in the eastern part of the Tarim Basin, at the junction of the Beishan terrane and eastern Tianshan fold belts (Fig. 1) where tectonic movements were strong and folds, fractures, joints, and fissures are well developed. In this area, the main fractures are the XingDi dextral fracture, Konqi River dextral fracture, Cheerchen sinistral fracture, and Altun sinistral fracture^21^. The study area is located in the northern Lop Nor basin (Fig. 1). Boundaries of each mining area and the brine storage layers are controlled by five faults^22^. The F1 tensional fault marks the border between the Luobei depression and Xinqing platform, with a strike of 30° and dip of approximately 120°, with linear extension. It rises in the west and forms a platform, while it sinks in the east and forms a depression. The F2 normal fault is located between the Tenglong platform in the east and the Luobei depression in the west, with a strike of 30° and dip of approximately 300°. The F5 normal fault is located in the eastern Tenglong platform, with a strike of 30° and dip of approximately 300°; it declines in the west to become a platform and uplifts in the east to become low mountains and hills. It also forms the line of demarcation between the Tenglong platform and the Beishan terrane. The F3 confined aquifer passes through the south-central Tenglong platform to divide the platform into a southern part and a northern part; it has a strike of 70° and a dip of 135°; the northern part of the fault sinks and the southern part uplifts. It controls the development of groundwater in the Tenglong platform. The F4 normal fault is located in the northern part of this area, and controls the northern boundaries of the three mining areas; with a strike of 100° and a dip of 190°^19,23^.

### Mining area brine elevation

From the GPS elevation data and brine buried depth measured on site, a contour map of mining area elevations was made with Surfer 11 (kriging) (Fig. 3). Underground brine in the Luobei depression is the lowest area, with minimum elevation of 775 m, maximum of 785 m, and average of 780 m; the brine elevation of the Xinqing platform is at the highest 795 m and the lowest 780 m, with an average of 789 m. The highest brine elevation of the Tenglong platform is 790 m and the lowest is 780 m, with an average of 785 m. The three mining areas are low in the middle and high on both sides. Because the Luobei depression is the lowest area, occasional transient surface runoff from the low surrounding hills or seasonal floods may occur and subsurface flow will converge here.

### Characteristics of the potassium-rich brine reservoir

**Figure 4:**
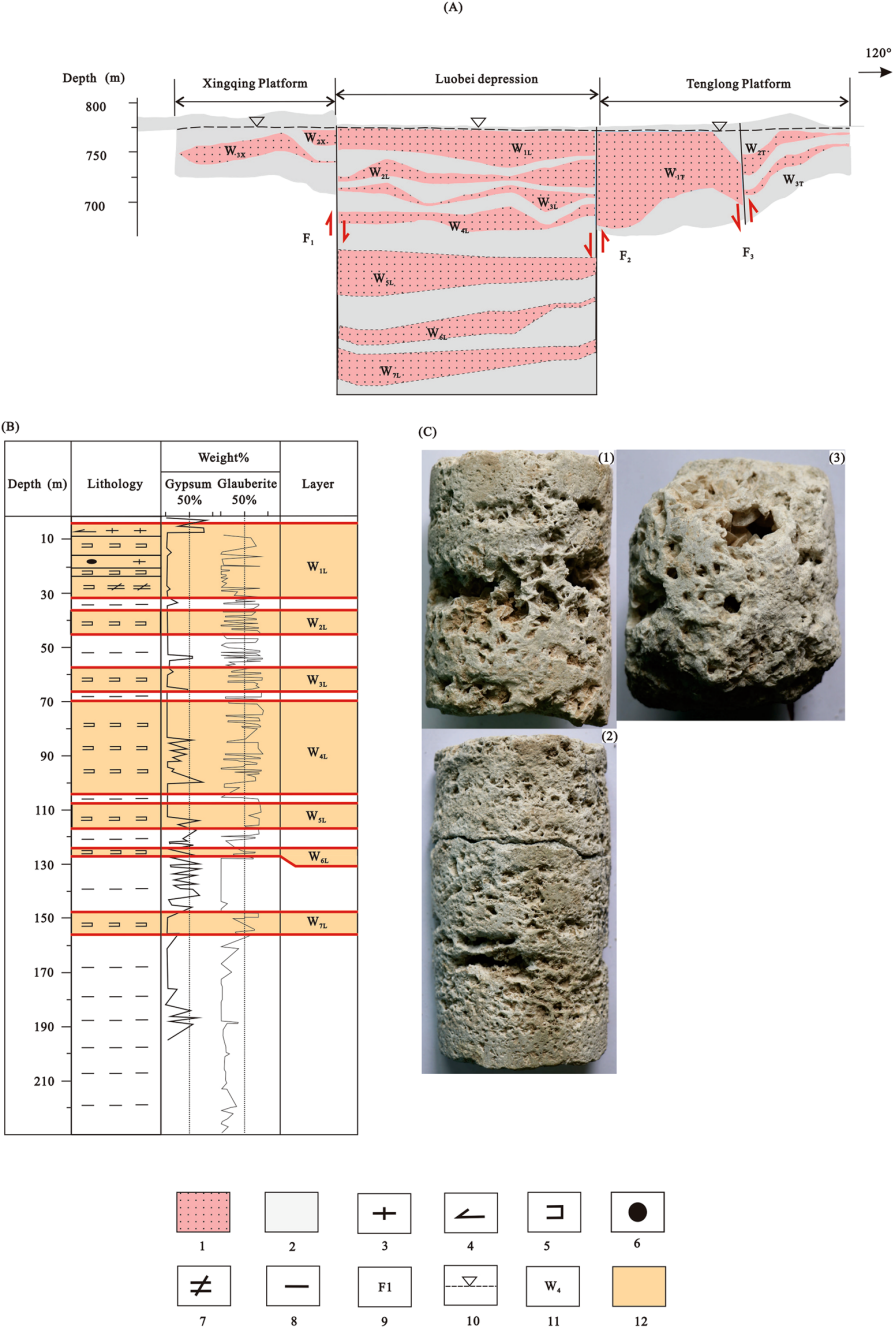
Vertical profile of brine reservoirs in each mining area. 1. glauberite reservoir; 2. clay water-resisting layer; 3. halite; 4. gypsum; 5. glauberite; 6. gravel; 7. bloedite; 8. clay; 9. buried fault; 10. equipotential line; 11. brine storage layer; 12. distribution of brine storage layer in drill hole. W_1L_ to W_7L_ are the storage layers in the Luobei depression; W_2X_ to W_3X_ are the storage layers in the Xinqing platform; and W_1T_ to W_3T_ are the storage layers in the Tenglong platform. (**A**) Vertical profiles of the three mining areas^23^; (**B**) lithological column with gypsum and glauberite contents of drill hole ZK1200 in the Luobei depression^6,28,29^; (**C**) photographs of glauberite pores, where (1), (2), and (3) represent depths of 10.5 m, 11.8 m, and 34.77 m, respectively.

The reservoir of the Lop Nor potassium-rich brine deposit is mainly glauberite with a gravel layer, but the existing and proven brine beds are mostly in glauberite layers. Only a few gravel strata contain potassium-rich brines, and their distribution and reserves are not clearly defined. Therefore, this paper only discusses glauberite reservoirs. Currently, 200 boreholes have been drilled in the Lop Nor mining area, and the Late-Middle Pleistocene to Late Pleistocene strata are distributed as a massive, continuous, thick layer of glauberite with well-developed intercrystal porosity (Fig. 4C), forming a good storage medium for potassium-rich brine. However, due to the influence of buried faults, there are differences in the brine storage layers among the three mining areas. There are seven brine beds (Fig. 4B) exposed by drill holes in the Luobei depression, including a phreatic aquifer, W_1L_, and six artesian aquifers, W_2L_, W_3L_, W_4L_, W_5L_, W_6L_, and W_7L_; however, due to the limitations of mining depth, at present, only W_1L_, W_2L_, W_3L_, and W_4L_ seams have been mined. There are two artesian brine aquifers, W_2X_ and W_3X_, exposed by drill holes in the Xinqing mining area^24^. There are three beds in the Tenglong mining area, including a phreatic aquifer, W_1T_, and two artesian aquifers, W_2T_ and W_3T_ (Fig. 4A).

#### Luobei depression mining area

The Luobei depression is the main storage site of the Lop Nor potassium-rich brine, with a north-south length of about 60 km, east-west width of 32.5 km, and a total area of 1411 km^2^ ^25^. There are stable salt-bearing clay aquiclude layers separating the seven brine layers in the mining area. The potassium-rich brines occur in glauberite rocks; lithologies of each brine storage layer with gypsum and glauberite contents can be seen in Fig. 4(B). W_1L_ is a phreatic aquifer with layered distribution across the whole Luobei depression, with average thickness of 17.54 m, water table depths of 1.7 to 2.3 m, porosities of 6.98% to 38.45%, and specific yields of 4.57% to 25.89%. Water yield is the highest in the central and northeast, with unit brine overflows of more than 5000 cubic meters per day per meter of water table depth (m^3^/d · m), while in the rest of the aquifer the unit brine overflows range from 1000 to 5000 m^3^/d · m^25^. The W_2L_ artesian aquifer is confined, nearly horizontal with a stratified distribution, and has an average thickness of 10.18 m, unit brine overflows of 10 to 100 m^3^/d · m, water table depths of 20 to 40 m, porosities of 4.34% to 37.8%, and specific yields of 1.08% to 21.04%. The W_3L_ artesian aquifer is confined, with stratified distribution and an average thickness of 8.50 m, unit brine overflows of 10 to 100 m^3^/d · m, water table depths of 40 to 70 m, porosities of 2.85% to 19.97%, and specific yields of 1.10% to 13.37%. The W_3L_ aquifer is also confined with stratified distribution, with an average thickness of 7.28 m, unit brine overflows of 10 to 100 m^3^/d · m, water table depths of 70 to 100 m, porosities of 5.22% to 24.72%, and specific yields of 1.03% to 9.91%^16^. The lithologies of the four brine storage layers are dominated by glauberite, and occasional lacustrine sedimentary clastic rocks, such as gypsum.

#### Xinqing platform mining area

The Xinqing platform consists of two confined potassium-bearing brine aquifers (Fig. 4A). Confined brines have layered or stratified distributions. The average thicknesses of the aquifers are 4.38 to 7.52 m. Due to control by the F1 fault, there is no phreatic aquifer in the Xinqing platform, but this does not affect the continuity of the brine storage layer between the mining areas. The W_2X_ aquifer is confined, stratified, and distributed in the eastern part of this ore district with a north-south length of 77.78 km, east-west width of 16.82 km, and total area of 1100 km^2^. Unit brine overflows are 2.25 to 541.51 m^3^/d · m, water table depths are 10 to 20 m, porosities are 3.89% to 40.69%, and specific yields are 2.01% to 21.15%. The W_3X_ aquifer is also confined and stratified, with a north-south length of 76.10 km, east-west width of 18.81 km, and total area of 1444 km^2^. Unit brine overflows are 1.67 to 293.99 m^3^/d · m, water table depths are 11.3 to 38 m, porosities are 4.16% to 26.43%, and specific yields are 2.11% to 14.19%^23^.

#### Tenglong platform mining area

The Tenglong platform consists of a phreatic aquifer and two confined aquifers. W_1T_ is a phreatic, stratified aquifer and is the main ore body, bounded by the F3 fault. It is distributed the northern part of the mining area, with a north-south length of about 33 km, east-west width of about 20 km, and total area of 610 km^2^. Water table depths are 3.26 to 4.6 m, porosities are 2.03% to 38.81%, and specific yields are 22.48% to 1.22%. On the other side of the F3 fault, in the southern part of the mining area, is the W_2T_ confined aquifer. Water table depths are 16.91 to 22 m, porosities are 3.58% to 37.64%, and specific yields are 1.35% to 18.69%. W_3T_ is also a confined aquifer, with a stratified orebody distributed in the southern part of the mining area, with a north-south length of about 29 km, east-west width of about 21 km, and total area of 546 km^2^. Water table depths are 17.13 to 47 m, porosities are 2.69% to 38.71%, and specific yields are 1.26% to 17.64%. Water table depths, porosities and specific yields for each brine storage layer are listed in Table 1.

**Table 1:** Water table depths, porosities, and specific yields for each brine storage layer in the Lop Nor potassium-rich brine deposit.

Mining area	aquifer	Water buried depth (m)	Porosity %	Specific yield %
Luobei depression	W_1L_	1.7–2.3	6.98–38.45%	4.57–25.89%
	W_2L_	20–40	4.34–37.8%	1.08–21.04%
	W_3L_	40–70	2.85–19.97%	1.10–13.37%
	W_4L_	70–100	5.22–24.72%	1.03–9.91%
Xinqing platform	W_2X_	10–20	3.89–40.69%	2.01–21.15%
	W_3X_	11.3–38	4.16–26.43%	2.11–14.19%
Tenglong platform	W_1T_	3.26–4.6	2.03–38.81%	22.48–1.22%
	W_2T_	16.91–22	3.58–37.64%	1.35–18.69%
	W_3T_	17.13–47	2.69–38.71%	1.26–17.64%

### Geochemical characteristics of potassium-rich brines

A total of 91 brine samples were collected from boreholes in the three mining areas, and analyzed for their chemical compositions, as listed in Table 2.

**Table 2:** Chemical compositions of potassium-rich brine in Lop Nor.

Mining area	Chemical composition	salinity	Ca^2+^	Mg^2+^	Na^+^	Cl^−^	SO_4_^−^	K^+^	HCO_3_	Br^−^	B^3+^	Li^+^
	unit	g/L	g/L	g/L	g/L	g/L	g/L	g/L	g/L	mg/L	mg/L	mg/L
Xinqing	max	356	0.8	20.1	101.7	186.1	5.9	8.8	0.2	35.2	724.5	18.1
	min	256	0	6.3	48.4	130.5	0.3	6.68	0.1	2.1	293.9	3.4
	average	332	0.2	11.2	80.9	167.9	2.3	7.2	0.2	15.1	478.5	11.2
	Coefficient of variation	31%	83%	31%	15%	8%	75%	13%	31%	48%	24%	29%
Luobei	max	385	0.4	29.29	99.2	194.7	7.35	9.8	0.3	29.3	715	25.3
	min	278	0.0	6.3	41.6	104.9	0.2	5.5	0.1	1.9	293.9	8.4
	average	367	0.2	13.6	81.3	176.3	2.5	8.0	0.2	12.1	504.2	16.8
	Coefficient of variation	26%	77%	36%	16%	8%	77%	12%	0.34%	61%	20%	23%
Tenglong	max	334	0.6	23.4	93.2	191.8	9.5	9.3	0.3	48.8	755.6	17.6
	min	247	0.0	7.8	24.4	102.8	0.2	6.1	0.0	2.5	277.3	8.9
	average	325	0.2	14.8	71.6	164.1	3.0	7.7	0.1	15.5	507.5	14.0
	coefficient of variation	24%	93%	31%	23%	11%	83%	12%	38%	65%	25%	15%

As described in Table 2, the brines of the three mining areas are all high salinity, ranging from 247 to 385 g/L. Luobei depression has the highest salinity, averaging 367 g/L, followed by Xinqing with an average of 332 g/L, and then Tenglong with an average of 325 g/L Cl^−^, and Na^+^ are the main components followed by Mg^+^, K^+^, and SO_4_^−^, while other elements are found in very small amounts and their abundances in the brine are not more than 1%. According to the classification system of Valyashko^26^ (1965), the Lop Nor brine deposit hydrochemistry is of the magnesium sulfate subtype.

The main targets for mining are potassium and boron. The K^+^ contents in the brines of the three ore districts range from 5.5 to 9.8 g/L, which are all higher than the industrial mining grade of KCl (1%)^27^. The average content of K^+^ is 7.2 g/L in the Xinqing mining area, 8.0 g/L in the Luobei mining area, and 7.7 g/L in the Tenglong mining area. The average coefficient of variation is 12.3%, indicating that the grade of potassium is stable. The content of B^3+^ in the three mining areas ranges from 277.3 to 755.6 mg/L, which are all higher than the comprehensive utilization grade of 150 mg/L, and in most brines the B^3+^ content is higher than the industrial mining grade of 300 mg/L^27^. Comprehensive development and use of brine can be carried out. The contents of bromine and lithium are generally low in the whole mining area, ranging from 1.9 to 48.8 mg/L for Br^−^ and from 3.4 to 25.3 mg/L for Li^+^, which are far lower than the comprehensive utilization grade of 150 mg/L for bromine and 13.1 mg/L for lithium. These latter two elements are not yet of mining value.

#### Spatial distribution trends of KCl contents

**Figure 5:**
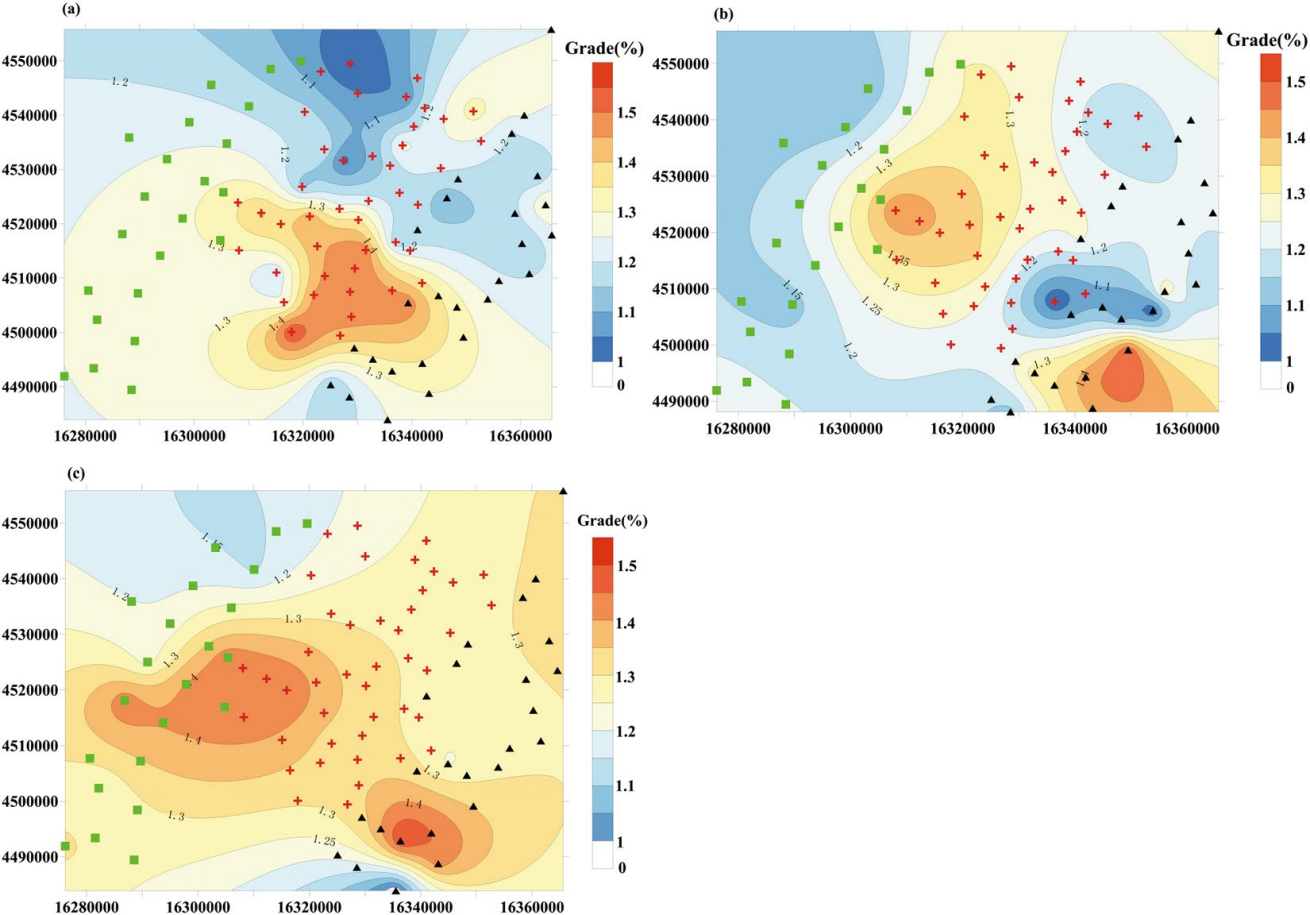
Distribution of KCl contents in different layers of potassium-rich brines at Lop Nor. (**a**) W_1_ reservoir; (**b**) W_2_ reservoir; (**c**) W_3_ reservoir.

We plotted isoline maps of KCl contents from measured results, using Surfer software (Fig. 5). The figure shows the spatial distributions of KCl contents for three layers (W_1_, W_2_, and W_3_) in the Lop Nor potassium brine deposit.

According to Fig. 5, the grades of KCl in the three ore beds are above the industrial mining grade (KCl content 1%) and the salinity and content of K^+^ of the brines increased with burial depth. The layers with KCl grades ≥1.2% make up more than 50% of the whole mining area, and as depth increases, the area increases. For the upper W_1_ layer, KCl contents of more than 1.2% in the brine are mainly found in the south-central part of the mining area, with the highest value 1.56%, and an average value of 1.3% (Fig. 5a). The contents of KCl in the middle, W_2_ ore layer, exhibit two high areas and one low area. The areas with more than 1.2% of KCl are mainly found in the north-central and southern regions of the mining area, with KCl contents of 1.2% to 1.45% and 1.2% to 1.55%, respectively. The average value is 1.21%. Between the two high value areas is a low value area, with KCl contents of 1% to 1.2% (Fig. 5b). In the W_3_ ore layer, the KCl grade is generally high, with KCl contents of more than 1.2% across most of the mining area, ranging from 1.2% to 1.5%, with an average of 1.31% (Fig. 5c).

The KCl grades in the three ore beds are more than the industrial grade. Since the Lop Nor potassium brine deposit was opened in 2004, it has been continually mined for 13 years. In comparison with the KCl grade identified before mining, the grade of KCl has decreased by 0.2% to 0.3%.

#### Spatial distribution trends of B_2_O_3_ contents

**Figure 6:**
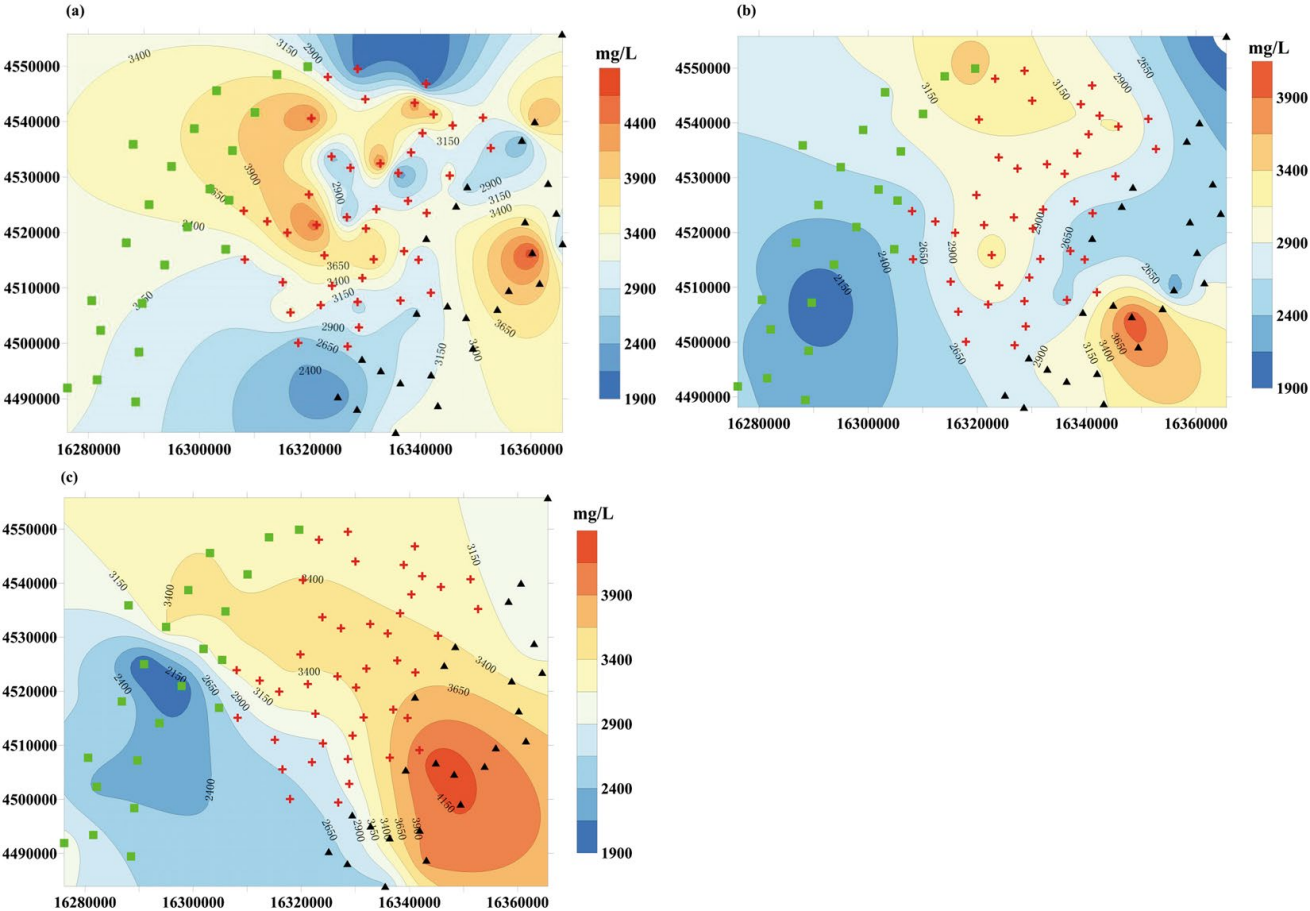
Distribution of B_2_O_3_ contents in potassium-rich brines at Lop Nor. (**a**) W_1_ reservoir; (**b**) W_2_ reservoir; (**c**) W_3_ reservoir.

The isoline maps(Fig. 6) of B_2_O_3_ contents in different ore beds indicate contents are higher than that of the industrial mining grade of 1000 mg/L, and that most B_2_O_3_ contents are more than 2400 mg/L. Content of B_2_O_3_ in the W_1_ reservoir (Fig. 6a) varies from 1900 to 4500 mg/L, with an average of 3200 mg/L. The highest value area (more than 3900 mg/L) is concentrated in the middle of the mining area. The content of B_2_O_3_ in the W_2_ reservoir (Fig. 6b) varies from 1900 to 4100 mg/L, with an average of 2900 mg/L. There are lower values in the northeastern and southwestern parts of the mining area and higher values in the northern and southeastern parts. The content of B_2_O_3_ in the W_3_ (Fig. 6c) reservoir ranges from 1900 to 4300 mg/L, with an average of 3000 mg/L. Higher values are distributed from the southeastern part of the mining area toward the northwest. Although the contents of B_2_O_3_ in the three layers are higher than that of the industrial mining grade, at present, only the potassium in the brine is mined.

## Conclusion

The Lop Nor brine deposit is a Quaternary continental saline lake, where a large amount of potassium-rich brine is contained in porous glauberite rock. This is very rare in Quaternary potassium basins, and the genesis of the deposit is still unclear. The deposit formed three mining areas under the control of tectonic action. These are, in order from west to east, the Xingqing platform, Luobei depression, and Tenglong platform. The Luobei depression is the main ore body, with the largest area and umber of storage aquifers (seven in the first 200 meters). However, only four layers, W_1L_ to W_4L_, are currently exploited. Potash reserves have not been ascertained for strata below 200 meters.

Lop Nor potassium-rich brine deposit contains high salinity brines, with salinity ranges of 247 to 385 g/L. KCl grade ranges from 1.2% to 1.45% with an average of 1.27%. B_2_O_3_ grade varies from 1900 to 4500 mg/L, which is higher than the industrial mining grade. The deposit is now mined by the SDIC Xinjiang Luobupo Hoevellite Co. Ltd and the main product is potassium sulfate, with an annual production capacity of 1.3 million tons. From the plotted KCl and B_2_O_3_ contour maps, an obvious desalination phenomenon is seen in the northern part of the W1 ore layer, possibly related to the low-salinity water submersible recharge in front of the Kulutage in the northern part of the basin. Therefore, it will be necessary to further exploit isotope tracer methods to construct models of the brine flow fields and take measures to ensure the quality and continued production of the deposit.
